# NTD and NCD Co-morbidities: The Example of Dengue Fever

**DOI:** 10.1371/journal.pntd.0004619

**Published:** 2016-08-25

**Authors:** Priyanka Mehta, Peter J. Hotez

**Affiliations:** 1 Rice University, Houston, Texas, United States of America; 2 Sabin Vaccine Institute and Texas Children’s Hospital Center for Vaccine Development, National School of Tropical Medicine at Baylor College of Medicine, Houston, Texas, United States of America; 3 Department of Biology, Baylor University, Waco, Texas, United States of America; 4 James A Baker III Institute for Public Policy, Rice University, Houston, Texas, United States of America; 5 Scowcroft Institute of International Affairs, Bush School of Government and Public Service at Texas A&M University, College Station, Texas, United States of America; Tropical Medicine Institute Pedro Kourí (IPK), CUBA

Recent findings of paradoxically high endemicity of neglected tropical diseases (NTDs) among populations living in the Group of 20 (G20) countries could portend high rates of these diseases among patients with underlying non-communicable diseases (NCDs), with resultant co-morbidities.

Today, the G20 countries, together with Nigeria, account for a surprising burden of the world’s NTDs, including approximately one-half of the major helminth infections and almost two-thirds or more of dengue, leprosy, Chagas disease, and leishmaniasis [1,2] as well as tuberculosis [3]. This finding has led to evidence of widespread neglected diseases among the poor who live in proximity to wealth, a concept that has been termed “blue marble health” [1–3].

Additional evidence that 70% of NCDs are also found in the G20 countries and Nigeria [4] suggests the possibility that we should find significant geographic overlap between NTDs and NCDs, especially in the largest middle-income countries such as Brazil, India, Indonesia, Mexico, and China. Ultimately, we can expect to see NTDs occurring among patients with underlying NCDs, and it will be of interest to determine whether unique co-morbidities arise as a result of such a clinical scenario.

## Dengue and NCDs

Dengue fever represents a potentially important example of an NTD now rapidly emerging or re-emerging among the G20 countries. According to the Global Burden of Diseases, Injuries, and Risk Factors 2013 study (GBD 2013), the incidence of dengue has increased an astounding 610.87% between 1990 and 2013 [5] with up to 390 million cases now occurring annually [6]. The largest number of cases of dengue is found in South Asia, especially India and Bangladesh, as well as in Southeast Asia—Indonesia, China, Philippines, Vietnam, Thailand—and in Brazil and Mexico in the Americas [6]. These same countries also account for some of the highest deaths resulting from NCDs, including diabetes, cardiovascular disease, cancer, and chronic obstructive pulmonary disease [4]. The literature on dengue co-morbidities with NCDs in Asia and the Americas is still in a relatively nascent stage, but it is growing. There is evidence that dengue exacerbates the effects of NCDs and vice versa (Box 1).

**Box 1. Major Co-morbidities Affecting Clinical Outcomes of Dengue Fever, DHF, and DHS**

**Underlying co-morbidities**
Diabetes Mellitus
Hypertension
Allergies (possibly associated with steroid use)

**Co-morbidities that complicate dengue**
Renal failure
Myocarditis/Pericarditis

## Diabetes and Hypertension: NCDs That Worsen Clinical Outcomes in Dengue Patients

In the Lahore dengue epidemic of 2011, 60% of dengue patients who died suffered from at least one co-morbid condition, especially diabetes mellitus and hypertension or diabetes with hypertension [7]. These same co-morbidities were found to be important risk factors for death in other dengue epidemics [8–11]. Indeed, these two NCDs stand out for their impact on dengue fever patients, including those with dengue shock syndrome (DSS) or dengue hemorrhagic fever (DHF).

### Diabetes

Dengue patients with pre-existing diabetes mellitus are reported 1.78 to 2.8 times more likely to develop DHF and tend to have more severe thrombocytopenia, which is often used as a measure of dengue infection severity [8,10,12,13]. Diabetes was associated with DHF and DSS during the epidemic in Havana, Cuba, in 1981 [14,15]. The physiologic mechanisms underlying this observation need to be further investigated. However, it has been suggested that patients with diabetes exhibit disrupted endothelial dysfunction, possibly because their altered metabolic state triggers the release of inflammatory cytokines—the resultant increase in endothelial permeability (vascular leak) may exacerbate third space fluid shifts characteristic of DHF [12,16]. As a result, dengue patients with co-morbid diabetes require longer hospitalizations and more medical attention than DHF patients without diabetes [8,10]. Uncontrolled diabetes is also associated with increased mortality; in a study from Kerala State, India, these patients are 26 times more likely to die as a result of their condition [11].

### Hypertension

A similar mechanism may explain why dengue patients with hypertension (or diabetes with hypertension) face a 1.6 to 2.16 times greater risk of progressing to DHF [10,17]. Hypertensive individuals often have elevated C-reactive protein levels in the blood, which increases capillary permeability and risk of coagulopathy [17]. In Kerala State, India, patients with underlying hypertension were found to be 44 times more likely to die from dengue [11].

### Other co-morbidities

Underlying chronic kidney disease and ischemic heart disease have also been linked to DHF and poor clinical outcome [8], although possibly these factors are also associated with diabetes and hypertension. Other interesting risk factors for severe dengue, DHF, or DSS include underlying allergies [12,17], autoimmune disorders [18], bronchial asthma [14], and sickle cell disease [14,19]. In at least one study, the steroids used to treat allergies might have been an important factor, as those patients exhibited a 2.94 odds ratio of DHF [12]; however, skin allergy itself was found to be a risk factor in a case-controlled study from six cities in Brazil [17].

## Renal Disease, Myocarditis, and Other Co-morbidities Arising from Dengue Fever

Conversely, certain co-morbidities arise as a result of dengue infection. One such disorder, acute renal failure (ARF), often occurs as a result of multi-organ dysfunction caused by severe forms of dengue, including DHF and DSS, occurring in approximately 2%–5% of the cases [20]. While most renal disorders associated with dengue are resolved without therapy, ARF significantly increases the chances of death [20]. Furthermore, in about 15% of patients, dengue was shown to cause cardiac disease (elevation of cardiac biomarkers), mostly with a clinical picture consistent with myocarditis or pericarditis [9,21], possibly due to direct viral invasion of the cardiac tissue [22]. Another condition noted to arise in association with dengue and DHF is a thickened gall bladder [8]. Some findings also suggest that worsening of chronic illness may be responsible for the high fatality rate seen in dengue patients with co-morbid conditions rather than worsening of the dengue itself [9]. In adult lymphoblastic leukemia patients, dengue infection often leads to febrile neutropenia and adds to the myelosuppression of chemotherapy, resulting in poor outcomes [23].

## Concluding Statement

Research on co-morbid conditions and their influence on the progression of dengue fever has yielded valuable insights. Reducing case fatality rates through management of risk factors, especially diabetes and hypertension, and dengue complications, especially renal and heart disease, provide important opportunities to improve clinical outcomes. Through heightened understanding of the complex interactions between dengue and several co-morbid NCDs, we can determine how to prioritize target populations for interventions and effectively allocate resources for treatment. A key example is the pipeline of dengue vaccines now under development [24], which could be introduced or prioritized for individuals with underlying diabetes and hypertension.

Dengue and its co-morbidities represent an important example of the overlap between NTDs and NCDs, especially among the poor. However, there is urgency to better understand co-morbidity pathogenesis in humans as well as to identify additional examples of NCD interactions among the 17 NTDs now recognized by the World Health Organization.
